# Latest advances and controversies of exercise therapy in the management of type 2 diabetes

**DOI:** 10.3389/fendo.2026.1822069

**Published:** 2026-04-01

**Authors:** Jiaman Hao, Huiyan Zhang

**Affiliations:** 1 Zhengzhou College of Finance and Economics, Zhengzhou, China; 2 Hainan Tropical Ocean University, Sanya, China; 3 New Era University, Selangor, Malaysia

**Keywords:** concurrent training, exercise therapy, high-intensity interval training, patient adherence, precision medicine, type 2 diabetes

## Abstract

Exercise therapy is a recognized cornerstone in the management of type 2 diabetes, offering broad benefits that extend beyond simple glycemic control to include cardiovascular and quality-of-life improvements. This mini-review synthesizes recent evidence on the efficacy of various exercise modalities, dosage optimization, and the ongoing tension between ideal clinical efficacy and real-world effectiveness. Recent meta-analyses indicate that high-intensity interval training (HIIT) is highly time-efficient and yields superior reductions in glycated hemoglobin (HbA1c). Meanwhile, concurrent training, which combines aerobic and resistance exercise, provides the most comprehensive metabolic and physiological benefits. Emerging research also emphasizes a paradigm shift toward personalized exercise prescriptions, suggesting an optimal physical activity dose of approximately 1100 MET-min/week that must be tailored to a patient’s baseline glycemic status. Despite clear physiological advantages, such as enhanced cardiorespiratory fitness and reduced chronic inflammation, long-term patient adherence remains a significant barrier to sustained effectiveness in real-world settings. Future clinical and research strategies must prioritize behavioral innovations to improve adherence, explore the synergistic effects of exercise with novel pharmacological treatments like GLP-1 receptor agonists, and foster sustainable, active lifestyles for patients with type 2 diabetes.

## Introduction

As a globally prevalent chronic metabolic disease, the management of type 2 diabetes has gone beyond simple glycemic control to a comprehensive strategy aimed at improving cardiovascular outcomes and enhancing the quality of life. Lifestyle interventions, particularly regular exercise, are recognized as the cornerstone of prevention and treatment. Abundant evidence indicates that exercise can effectively improve insulin sensitivity, reduce blood glucose, and provide broad metabolic benefits (1). However, although the “efficacy” of exercise therapy has been repeatedly validated in strictly controlled research settings, its long-term “effectiveness” in the real world remains highly controversial due to adherence challenges (2). A central dilemma in contemporary clinical practice is how to develop personalized, highly efficient, and sustainable exercise prescriptions, given the wide array of available modalities, such as high-intensity interval training (HIIT) and concurrent training, and the significant heterogeneity among patients with diabetes. This tension between ideal “efficacy” and realistic “effectiveness” constitutes the focus of current research exploration and controversy.

This mini-review aims to summarize the latest evidence on exercise therapy for type 2 diabetes, with a focus on comparing the effects of different exercise modalities, exploring dosage optimization strategies, and examining the comprehensive benefits beyond glycemic control as well as existing controversies.

This article aims to provide a narrative mini-review rather than a systematic review. The literature discussed is based on a targeted search of major biomedical databases, including PubMed and Web of Science, focusing on studies published between January 2024 and December 2025. Priority was given to recent systematic reviews, meta-analyses, umbrella reviews, and selected original studies deemed to offer clinical or mechanistic insights into exercise therapy for type 2 diabetes. As this review is not a formal systematic review, its evidence synthesis should be interpreted as a focused and clinically oriented overview, not an exhaustive evaluation of all available literature.

## Comparison of the efficacy of exercise modalities on glycemic control: from HIIT and concurrent training to traditional aerobic and resistance exercise

The choice of exercise modality is a key variable influencing efficacy. In recent years, high-intensity interval training has received widespread attention for its time efficiency. Several recent meta-analyses have confirmed that HIIT demonstrates significant advantages in improving glycated hemoglobin (HbA1c) in patients with type 2 diabetes (3, 4). A network meta-analysis including 85 randomized controlled trials indicated that HIIT had the greatest effect on reducing HbA1c (MD = -0.78%), followed by concurrent training, yoga, and continuous aerobic exercise, although the differences in efficacy between the modalities were not statistically significant (3). Another umbrella review also supports that HIIT is not only superior to non-exercising control groups in improving HbA1c and cardiorespiratory fitness, but it is also slightly superior to traditional moderate-intensity continuous training (4). Specifically, compared with the control group, HIIT can reduce HbA1c by 0.75% and can effectively improve fasting blood glucose, 2-hour postprandial blood glucose, and the insulin resistance index (5, 6).

Concurrent training, which combines aerobic and resistance exercise within the same training session, is considered a highly potential modality because it can simultaneously target multiple physiological systems. Studies consistently show that concurrent training can significantly improve HbA1c, fasting blood glucose, insulin resistance, and the lipid profile (7–9). A Bayesian dose-response meta-analysis demonstrated that concurrent training can significantly reduce HbA1c levels (MD = -0.48%) (10). Network meta-analysis results suggest that concurrent training may have the greatest magnitude of HbA1c reduction (-0.74%) (11). Compared with a single modality, the synergistic effect of concurrent training has been demonstrated in some studies. For instance, in patients with type 2 diabetes, the combination of exercise and metformin treatment showed a trend toward better improvement in HbA1c and fasting blood glucose than either treatment used alone (1).

Traditional aerobic exercise and resistance exercise remain important foundations. Continuous aerobic exercise can effectively reduce 24-hour mean blood glucose (12). Resistance training, in addition to improving blood glucose, has been proven to reduce inflammatory markers such as C-reactive protein and exert a positive impact on various indicators of metabolic syndrome (e.g., blood pressure, waist circumference, and blood lipids) (13, 14). It should be noted that the effects of different exercise modalities may vary depending on the stage of the disease. A network meta-analysis found that in the prediabetic population, the effects of exercise on improving HbA1c, 2-hour postprandial blood glucose, and insulin resistance were superior to metformin; however, in patients with established type 2 diabetes, the glucose-lowering efficacy of metformin appeared to be superior to exercise alone (1). This highlights the impact of disease severity on the selection of intervention measures. At the same time, these comparative findings should be interpreted with appropriate caution. Much of the current evidence base is derived from meta-analyses and umbrella reviews that pool studies with substantial differences in exercise modality, intensity, duration, supervision, comparator groups, baseline glycemic status, and outcome definitions. In addition, overlap among the underlying primary trials across pooled analyses may limit the apparent independence of the evidence. Accordingly, although the overall direction of benefit is consistent, direct comparisons between exercise modalities should not be viewed as definitive in the absence of more standardized and methodologically harmonized primary trials.

## Optimization of exercise dosage: personalized prescriptions based on baseline glycemic status and dose-response relationships

The “optimal exercise dose” has always been a hotspot and a difficulty in research. Recent evidence is driving a shift in exercise prescriptions from a “one-size-fits-all” approach to personalization. A Bayesian meta-analysis based on 126 high-quality studies proposed, for the first time, precise dosage recommendations considering baseline HbA1c levels: for patients with different glycemic control statuses, the physical activity dose required to achieve the maximum HbA1c reduction effect is 1100 MET-min per week (approximately equivalent to 300 minutes of moderate-intensity activity per week). However, at this dose, the expected reduction varies among populations with different baselines: -1.02% to -0.66% for severely uncontrolled diabetes, -0.64% to -0.49% for uncontrolled diabetes, -0.47% to -0.40% for controlled diabetes, and -0.38% to -0.24% for prediabetes (15). This provides a crucial basis for clinicians to set realistic and personalized improvement goals based on the patient’s initial blood glucose level.

Research on dose-response relationships has further revealed the non-linear characteristics of exercise benefits. One meta-analysis found that a significant improvement in HbA1c levels can be observed when the total exercise dose reaches 840 MET-min/week (3). Another Bayesian analysis focusing on concurrent training similarly confirmed a non-linear dose-response relationship between concurrent training and HbA1c, with an optimal dose of approximately 1030 MET-min/week (10). This suggests the presence of a “threshold effect,” where an exercise volume below this threshold may fail to produce significant metabolic improvements.

In addition to the total volume, the specific parameter configuration of the exercise regimen also affects the outcome. For HIIT, longer durations (e.g., >30 seconds/interval), higher training volumes, and medium-to-long-term (>4 weeks) interventions appear to yield better improvements in body composition, glycemic control, and cardiorespiratory fitness (5, 16). For traditional exercise, supervised interventions and an exercise volume of 150–210 minutes per week have proven to be the most effective (11). Furthermore, traditional Chinese exercises such as Tai Chi and Ba Duan Jin have also been proven effective, with a recommended regimen of 30–50 minutes per session, 4–5 times a week, lasting for at least 3 months (17). These findings collectively point to a core principle: an effective exercise prescription must comprehensively consider the exercise modality, intensity, frequency, duration, and the individual baseline status of the patient. This also underscores an important clinical reality: the optimal exercise dose is unlikely to be identical for all individuals with type 2 diabetes. Differences in age, disease duration, baseline HbA1c, obesity status, cardiovascular comorbidity, medication use, physical limitations, and behavioral readiness may all influence both physiological response and long-term adherence. For this reason, dose optimization should be understood not only as a question of maximizing short-term metabolic efficacy, but also as a process of tailoring exercise prescriptions to patient-specific capacity, risk profile, and real-world feasibility.

The key characteristics, glycemic effects, and broader physiological benefits of major exercise modalities are summarized in **Table 1**.

**Table 1:** Summary of exercise modalities and their clinical benefits in type 2 diabetes management.

Exercise Modality	Key Characteristics and Optimal Parameters	Primary Glycemic Effects	Comprehensive Physiological Benefits
High-Intensity Interval Training (HIIT)	High time efficiency: longer durations (>30 s/interval) and >4 weeks of intervention are associated with better outcomes.	Greatest reduction in HbA1c (MD up to -0.78%); improves fasting and 2-h postprandial glucose.	Improves peak oxygen uptake (VO2 peak), left ventricular mass, and ejection fraction.
Concurrent Training (Aerobic + Resistance)	The optimal dose is approximately 1030 MET-min/week.	Significant HbA1c reduction (-0.48% to -0.74%); improves fasting blood glucose and insulin resistance.	Synergistic effects across multiple systems improve lipid profile and overall cardiometabolic health.
Continuous Aerobic Training	Optimal volume of 150–210 minutes per week; often performed at moderate intensity.	Effectively reduces 24-hour mean blood glucose levels.	Increases left ventricular end-diastolic volume; most effective for improving flow-mediated vasodilation.
Resistance Training	Focuses on muscle strengthening; synergistic when combined with aerobic exercise.	Contributes to overall glycemic control.	Specifically reduces C-reactive protein (CRP); improves metabolic syndrome indicators (blood pressure, waist circumference, blood lipids).
Traditional Chinese Exercises	Tai Chi, Ba Duan Jin, etc. Recommended: 30–50 min/session, 4–5 times/week for >= 3 months.	Effective in improving overall glycemic control.	Suitable for patients with exercise intolerance; promotes physical and mental well-being.

## Beyond blood glucose: comprehensive benefits and controversies of exercise on cardiometabolic health, inflammation, and quality of life

The value of exercise therapy goes far beyond lowering blood glucose. Patients with type 2 diabetes often suffer from exercise intolerance, with their peak oxygen uptake significantly lower than that of healthy populations, which is correlated with abnormal diastolic function indicators and the duration of diabetes (18). Exercise, particularly HIIT and concurrent training, can effectively increase peak oxygen uptake and improve cardiorespiratory fitness (4, 19). In terms of cardiac structure and function, both HIIT and moderate-intensity continuous training can increase left ventricular end-diastolic volume, while HIIT may have a greater advantage in improving left ventricular mass and ejection fraction (19). Vascular endothelial dysfunction is an early marker of cardiovascular complications, and aerobic interval exercise is considered by a network meta-analysis to be the most effective modality for improving flow-mediated vasodilation (20). From a clinical perspective, this broader framing is important because the value of exercise therapy in type 2 diabetes should not be judged solely by HbA1c reduction. Although glycemic control remains a central therapeutic target, exercise may also confer meaningful benefits in cardiovascular risk, cardiorespiratory fitness, endothelial function, functional capacity, inflammatory burden, quality of life, and other patient-centered outcomes. In some patients, these broader benefits may be clinically important even when improvements in glycemic indices appear modest.

The regulatory effect of exercise on chronic low-grade inflammation and oxidative stress has also attracted considerable attention. Overall, exercise training can effectively reduce the levels of interleukin-6, tumor necrosis factor-α, C-reactive protein, and leptin, while increasing adiponectin levels (21). However, the effects vary across different exercise modalities. Aerobic training and concurrent aerobic and resistance training have shown good effects in improving various inflammatory markers, whereas the impact of pure resistance training or HIIT on some inflammatory indicators has not reached significance (21). Resistance training has been proven to specifically reduce C-reactive protein (13). Simultaneously, exercise can also reduce the oxidative stress marker malondialdehyde and increase superoxide dismutase activity (22).

Although the aforementioned physiological benefits are clear, translating these benefits into perceptible improvements in quality of life and maintaining them long-term in the real world remains challenging. Comprehensive multi-component exercise interventions have been shown to significantly enhance physical strength, physiological function, mental health, and overall health perception in patients with type 2 diabetes (7). Diabetes self-management education has also been proven to effectively improve patients’ self-management capabilities and quality of life. However, a point of controversy is whether the promotion of behavioral changes through lifestyle interventions can be stably translated into significant improvements in clinical outcomes. A meta-analysis found that while lifestyle interventions aimed at promoting physical activity can slightly increase daily activity time, their effect on lowering HbA1c is very limited (-0.09%), and the increase in physical activity itself did not demonstrate a significant moderating effect on changes in HbA1c (23). This reveals that there may be complex intermediate links and individual differences between “increasing activity levels” and “improving blood glucose”. The greatest controversy and challenge lie in long-term adherence. From an evolutionary perspective, exercise is a counter-instinctual behavior, making it particularly difficult to sustain long-term for patients with type 2 diabetes who already experience discomfort. To date, no studies have been able to confirm that patients’ adherence to structured exercise programs can be sustained for more than a year (2), which severely restricts the long-term effectiveness of exercise as “medicine”.

Nevertheless, the interpretation of these broader benefits also requires caution. Much of the available synthesis relies on secondary evidence, particularly systematic reviews, meta-analyses, and umbrella reviews, which may aggregate studies of varying methodological quality and substantial clinical heterogeneity. Differences in participant characteristics, disease severity, intervention design, follow-up duration, and endpoint selection can all influence pooled estimates. Furthermore, repeated inclusion of the same primary studies across different reviews may exaggerate the perceived consistency of the evidence base. A more rigorous next generation of trials with harmonized outcomes and clearer phenotypic stratification will therefore be important for refining the clinical interpretation of these findings.

## Conclusion and future directions

In summary, recent evidence has further consolidated the status of exercise as a foundational treatment for type 2 diabetes. High-intensity interval training demonstrates efficient time benefits, concurrent training provides the broadest comprehensive benefits, and personalized dosage prescriptions based on baseline HbA1c levels represent a new direction in precision sports medicine. The multiple benefits of exercise in improving cardiovascular function, reducing inflammation, and enhancing quality of life have been widely recognized.

Looking ahead, research needs to shift from proving short-term efficacy to solving the fundamental problem of long-term effectiveness. First, intervention strategies must be innovated to improve long-term adherence, such as integrating exercise into daily life, utilizing social media support, or designing more engaging programs based on behavioral change theories. Second, exploring the synergistic effects of exercise and emerging therapies (such as GLP-1 receptor agonists) is crucial (24). Preliminary views suggest that combination pharmacotherapy and lifestyle changes (especially increasing protein intake and strength training) may become the mainstream direction of future obesity and diabetes management, aiming to mitigate muscle loss and consolidate long-term efficacy. Furthermore, more mechanistic studies are needed to elucidate the biological pathways through which different exercise modalities produce specific benefits, as well as the impact of individual differences (e.g., genotypes, phenotypes) on exercise responses. In this review, specific attention should be given to the cardiovascular effects of exercise at the level of myocardial mechanics. Global longitudinal strain and other strain-derived indices are increasingly recognized as sensitive markers of subclinical diabetic cardiomyopathy, as they are frequently impaired in individuals with diabetes despite a preserved left ventricular ejection fraction (25, 26). Future randomized or longitudinal studies evaluating the impact of structured exercise programs (e.g., high-intensity interval training or concurrent training) on myocardial deformation parameters assessed by speckle-tracking echocardiography could provide valuable insights into early cardiac remodeling and cardiovascular risk modification. Finally, clinical practice should promote a paradigm shift from “prescribing exercise” to “co-creating sustainable active lifestyles,” integrating multidisciplinary resources such as medicine, sports science, and psychology to develop truly personalized, feasible, and resilient lifelong health management strategies for patients with type 2 diabetes.

Overall, exercise therapy in type 2 diabetes should be understood not merely as a glucose-lowering strategy but as a multidimensional clinical intervention whose value extends to cardiovascular health, physical function, and patient well-being. Its future impact will depend not only on physiological efficacy but also on transparent evidence synthesis, individualized prescription, and the development of practical strategies that support long-term implementation in real-world care.
